# Supplementary material for: Heat adaptation of phage T7 under an extended genetic code
Source: Virus Evol. 2021 Dec 1;7(2):veab100. doi: 10.1093/ve/veab100 (PMC8923235; doi:10.1093/ve/veab100)
Supplement: veab100_Supp [file veab100_supp.zip › Sequencing_Analysis.zip!/Sequencing_Analysis/A1/evidence/JC_51.html]

BRESEQ :: Evidence


| New junction evidence | | | | | | | | | | | |
| --- | --- | --- | --- | --- | --- | --- | --- | --- | --- | --- | --- |
|  | | seq id | position | reads (cov) | reads (cov) | score | skew | freq | annotation | gene | product |
| \* | ? | NC\_001604.1 | 295 = | 3899 (1.260) | 25 (0.010) | 15/560 | 36.7 | 0.68% | intergenic (–/–) | *–/–* | –/– |
|  | ? | NC\_001604.1 | = 281 | 3655 (1.270) |  |  |  |  | intergenic (–/–) | *–/–* | –/– |
| Rejected: Coverage evenness skew score above cutoff. | | | | | | | | | | | |
| Rejected: Frequency below/above cutoff threshold. | | | | | | | | | | | |

Only 100 of 439 total aligned reads displayed.  

`TACTGCATGTTACTTCATGTTGTCAATACCTGTTTTTCGTGCGACTTATCAGGCTGTCTACTTATCCGGTTGACCCCGGTGTCGATTGGGATGGCTATTCGCCGTGTCCCTCTCGATGGCTGTAAGTATCCTATAGGTTAGACTTTAAGTCAATACTCTTTTTGATAAATTTTAAATTAATCTTTTAAGTCTCTTTAAGTTGTCTCTCCTTATAGTGAGTTGTATTAATAAAGACAATGATTAAAGGTTATCACTTTATGGCTAACAACAAGGCGTCTTTAGGTCTGGTCTTTATGTAGTGTCTTTAGGTCTGGTCTTTA‑‑‑‑‑‑‑‑‑‑‑‑‑‑‑‑‑‑‑‑‑‑‑‑‑‑‑‑‑‑‑‑‑‑‑‑‑‑‑‑‑‑‑‑‑‑‑‑‑‑‑‑‑‑‑‑‑‑‑‑‑‑‑‑‑‑‑‑‑‑‑‑‑‑‑‑‑‑‑‑‑‑‑‑‑‑‑‑‑‑‑‑‑‑‑‑‑‑‑‑‑‑‑‑‑‑‑‑‑‑‑‑‑‑‑‑‑‑‑‑‑‑‑‑‑‑‑‑‑‑‑‑‑‑‑‑‑‑‑‑‑‑‑‑‑‑‑‑‑‑‑‑‑‑‑‑‑‑‑‑‑‑‑‑‑‑‑‑‑‑‑‑‑‑‑‑‑‑‑‑‑‑‑‑‑‑‑‑‑‑‑‑‑‑‑‑‑‑‑‑‑‑‑‑‑‑‑‑‑‑‑‑‑‑‑‑‑‑‑‑‑‑‑‑‑‑‑‑‑‑‑‑‑‑‑‑‑‑‑‑‑‑‑‑‑‑‑‑‑‑‑‑‑‑‑‑‑‑‑‑‑‑‑‑‑‑‑‑‑‑‑‑‑‑‑‑‑‑‑‑‑‑‑‑‑‑‑‑‑‑‑‑‑‑‑‑‑‑‑‑‑‑  <  NC_001604.1/614‑295`  
`‑‑‑‑‑‑‑‑‑‑‑‑‑‑‑‑‑‑‑‑‑‑‑‑‑‑‑‑‑‑‑‑‑‑‑‑‑‑‑‑‑‑‑‑‑‑‑‑‑‑‑‑‑‑‑‑‑‑‑‑‑‑‑‑‑‑‑‑‑‑‑‑‑‑‑‑‑‑‑‑‑‑‑‑‑‑‑‑‑‑‑‑‑‑‑‑‑‑‑‑‑‑‑‑‑‑‑‑‑‑‑‑‑‑‑‑‑‑‑‑‑‑‑‑‑‑‑‑‑‑‑‑‑‑‑‑‑‑‑‑‑‑‑‑‑‑‑‑‑‑‑‑‑‑‑‑‑‑‑‑‑‑‑‑‑‑‑‑‑‑‑‑‑‑‑‑‑‑‑‑‑‑‑‑‑‑‑‑‑‑‑‑‑‑‑‑‑‑‑‑‑‑‑‑‑‑‑‑‑‑‑‑‑‑‑‑‑‑‑‑‑‑‑‑‑‑‑‑‑‑‑‑‑‑‑‑‑‑‑‑‑‑‑‑‑‑‑‑‑‑‑‑‑‑‑‑‑‑‑‑‑‑‑‑‑‑‑‑‑‑‑‑‑‑‑‑‑‑‑‑‑‑‑‑‑‑‑‑‑‑‑‑‑‑‑‑‑‑‑‑gtctttaggtctggtctttaTGTTTAAACTTTAAGATAGGCGTTGACTTGATGGGTCTTTAGGTGTAGGCTTTAGGTGTTGGCTTTAGGATGGACGTTAGGAGGTGACTTTAGGAGGATACTTTAGGAGACTGTAACAGATAGGGACACAGAGAGACACTCAAGGTAACACCCAAAGACAAACCCAAGGGTCATCCCCAACCCTTAGGGAACCCTCAAGGTCAACCGAAGGTTGACTTTAGGTGATTGGCTGGGCTTTAGGTACCCCCTATGGGGGAACTTTAGGTCCGTACACTGTG‑‑‑‑‑‑‑‑‑‑‑‑‑‑‑‑‑‑‑‑‑AGA  <  NC_001604.1/281‑1`  

                                                                                                                                                                                                                                                                                                                                                                                                                                                                                                                                                                                                                                                
`TACTGCATGTTACTTCATGTTGTCAATACCTGTTTTTCGTGCGACTTATCAGGCTGTCTACTTATCCGGTTGACCCCGGTGTCGATTGGGATGGCTATTCGCCGTGTCCCTCTCGATGGCTGTAAGTATCCTATAGGTTAGACTTTAAGTCAATACTCTTTTTGATAAATTTTAAATTAATCTTTTAAGTCTCTTTAAGTTGTCTCTCCTTATAGTGAGTTGTATTAATAAAGACAATGATTAAAGGTTATCACTTTATGGCTAACAACAAGGCGTCTTTAGGTCTGGTCTTTATGTAGTG                                                                                                                                                                                                                                                                                                                                   >  1:167636/1‑301`  
`TACTGCATGTTACTTCATGTTGTCAATACCTGTTTTTCGTGCGACTTAGCAGGCTGTCTACTTATCCGGTTGACCTCGGTGTAGATTGGGATGGCTATTCGCCGTGTCCCTCTCGATGGCTGTAAGTATCCTATAGGTTAGACTTTAAGTCAATACTCTTTTTGATAAATTTTAAATTAATCTTTTAAGTCTCTTTAAGTTGTCTCTCCTTATAGTGAGTTGTATTAATAAAGACAATGATTAAAGGTTATCACTTTATGGCTAACAACAAGGCGTCTTTAGGTCTGGTCTTTATGTAGTG                                                                                                                                                                                                                                                                                                                                   <  1:140726/301‑1`  
 `ACTGTATGTTACTTCTTTTTGTCAATACCTTTTTTTCGTGCGACTTATCAGGCTGTCTACTTATCCGGTTGACCCCTGTGTCGATTGGGATGGCTATTCGCCGTGTCCCTCTCGATGGCTGTAAGTATCCTATAGGTTAGACTTTAAGTCAATACTCTTTTTGATAAATTTTAAATTAATCTTTTAAGTCTCTTTAAGTTGTCTCTCCTTATAGTGAGTTGTATTAATAAAGAAAATGATTAAAGGTTATCACTTTATGGCTAACAACAAGGCGTCTTTAGGTCTGGTCTTTATGTAGTGT                                                                                                                                                                                                                                                                                                                                  <  1:506019/301‑1`  
 `ACTGCATGTTACTTTATGTTGTCAATACCTGTTTTTCGTGCGACTTATCAGGCTGTCTACTTATCCGGTTGGCCCCGGTGTCGATTGGGATGGCTATTCGCCGTGTCCCTCTCGATGGCTGTAAGTATCCTATAGGTTAGACTTTAAGTCAATACTCTTTTTGATAAATTTTAAATTAATCTTTTAAGTCTCTTTAAGTTGTCTCTCCTTATAGTGAGTTGTATTAATAAAGACAATGATTAAAGGTTATCACTTTATGGCTAACAACAAGGCGTCTTTAGGTCTGGTCTTTATGTAGTGT                                                                                                                                                                                                                                                                                                                                  <  1:431312/301‑1`  
 `ACTGCATGTTACTTCATGTTGTCAATACCTGTTTTTCGTGCGACTTATCAGGCTGTCTACTTATCCGGTTGACCCCGGTGTCGATTGGGATGGCTATTCGCCGTGTCCCTCTCGATGGCTGTAAGTATCCTATAGGTTAGACTTTAAGTCAATACTCTTTTTGATAAATTTTAAATTAATCTTTTAAGTCTCTTTAAGTTGTCTATCCTTATAGTGAGTTGTATTAATAAAGACAATGATTAAAGGTTATCACTTTATGGCTAACAACAAGGCGTCTTTAGGTCTGGTCTTTATGTAGTGT                                                                                                                                                                                                                                                                                                                                  >  1:197342/1‑301`  
 `ACTGCATGTTACTTCATGTTGTCAATACCTGTTTTTCGTGCGACTTATCAGGCTGTCTACTTATCCGGTTGACCCCGGTGTCGATTGGGATGGCTATTCGCCGTGTCCCTCTCGATGGCCGTAAGTATCCTAAAGGTTAGACTTTAAGTCAATACTCTTTTTGATAAATTTTAAATTAATCTTTTAAGTCTCTTTAAGTTGTCTCTCCTTATAGTGAGTTGTATTAATAAAGACAATGATTAAAGGTTATCACTTTATGGCTAACAACAAGGCGTCTTTAGGTCTGGTCTTTATGTAGTGT                                                                                                                                                                                                                                                                                                                                  <  1:420807/301‑1`  
  `CTGCATGTTACTTCATGTTGTCAATACCTGTTTTTCGTGCGACTTATCAGGCTGTCTACTTATCCGGTTGACCCCGGTGTCGATTGGGATGGCTATTCGCCGTGTCCCTCTCGATTGCTGTAAGTATCCTATAGGTTAGACTTTAAGTCAATACTCTTTTTGATAAATTTTAAATTAATCTTTTAAGTCTCTTTAAGTTGTCTCTCCTTATAGTGAGTTGTATTATTAAATACAATGATTAAAGGTTATCATTTTATGGCTAACAACAAGGCGTCTTTAGGTCTGGTCTTTATGTAGTGTt                                                                                                                                                                                                                                                                                                                                 >  1:22223/1‑300`  
  `CTGCATGTTACTTCATGTTGTCAATACCTGTTTTTCGTGCGACTTATCAGGCTGTCTACTTATCCGGTTGACCCCGGTGTCGATTGGGATGGCTATTCGCCGTGTCCCTCTCGATGGCTGTAAGTATCCTATAGGTTAGACTTTAAGTCAATACTCTTTTTGATAAATTTTAAATTAATCTTTTAAGTCTCTTTAAGTTGTCTCTCCTTATAGTGAGTTGTATTAATAAAGACAATGATTAAAGGTTATCACTTTATGGCTAACAACAAGGCGTCTTTAGGTCTGGTCTTTATGTAGTGTC                                                                                                                                                                                                                                                                                                                                 <  1:20109/301‑1`  
   `TGCATGTTACTTCATGTTGTCAATACCTGTTTTTCGTGCGACTTATCAGGCTGTCTACTTATCCGGTTGACACCGGTGTCGATTGGGATGGCTATTCGCCGTGTCCCTCTCGATGGCTGTAAGTATCCTATAGGTTAGACTTTAAGTAAATACTCTTTTTGATAAATTTTAAATTAATCTTTTAAGTCTCTTTAAGTTGTCTCTCCTTATAGTGAGTTGTATTAATAAAGACAATGATTAAAGGTTATCACTTTATGGCTAACAACAAGGCGTCTTTAGGTCTGGTCTTTATGTAGTGTCT                                                                                                                                                                                                                                                                                                                                <  1:347008/301‑1`  
    `GCATGTTACTTCATGTTGTCAATACCTGTTTTTCGTGCGACTTATCAGGCTGTCTACTTATCCGGTTGACCCCGGTGTCGCTTGGGATGGCTATTCGCCGTTTCCCTCTGGATGGCTGTATGTATCCTATAGGTTAGACTTTAAGTCACTACTCTTTTTGATAAATTTTAAATTAATCTTTTAAGTCTCTTTAAGTTGTCTCTACTTATAGTTAGTTGTATTAATAAAGACTATGATTAAAGGTGTTCTCTTTATGGCTAAAAACAAGGTGTCTTTAGGTCTGGTCTTTATGTATTGTCTT                                                                                                                                                                                                                                                                                                                               >  1:41139/1‑301`  
    `GCATGTTACTTCATGTTGTCAATACCTGTTTTTCGTGCGACTTATCAGGCTGTCTACTTATCCGGTTGACCCCGGTGTCGATTGGGATGGCTATTCGTCGTGTCCCTCTCGATGGCTGTAAGTATCCTATAGGTTAGACTTTAAGTCAATACTCTTTTTGATAAATTTTAAATTAATCTTTTAAGTCTCTTTAAGTTGTCTCTCCTTATAGTGAGTTGTATTAATAAAGACAATGATTAAAGGTTATCACTTTATGGCTAACAACAAGGCGTCTTTAGGTCTGGTCTTTATGTAGTGTCTT                                                                                                                                                                                                                                                                                                                               >  1:122574/1‑301`  
    `GCATGTTACTTCATGTTGTCAATACCTGTTTTTCGTGCGACTTATCAGGCTGTCTACTTATCCGGTTGACCCCGGTGTCGATTGGGATGGCTATTCGCCGTGTCCCTCTCGATGGCTGTAAGTATCCTATCGGTTAGACTTTAAGTCAATACTCTTTTTGATAAATTTTCAATTAATCTTTTAAGTCTCTTTAAGTTGTCTCTCCTTATAGTGAGTTGTATTACTAAAGACAATGATTAAAGGTTATCACTTTATGGCTAACAACAAGGCGTCTTTAGGTCTGGTCTTTATGTAGTGTCTT                                                                                                                                                                                                                                                                                                                               >  1:81640/1‑301`  
    `GCATGTTACTTCATGTTGTCAATACCTGTTTTTCGTGCGACTTATCAGGCTGTCTACTTATCCGGTTGACCCCGGTGTCGATTGGGATGGCTATTCGCCGTGTCCCTCTCGATGGCTGTAAGTATCCTATAGGTTCGACTTTAAGTCAATACTCTTTTTGATAAATTTTAAATTAATCTTTTAAGTCTCTTTAAGTTGTCTCTCCTGATAGTGAGTTGTATTCATAAAGACAATGATTAAAGGTTATCACTTTATGGCTAACAACAAGGCGTCTTTAGGTCTGGTCTTTATGTAGTGTCTT                                                                                                                                                                                                                                                                                                                               >  1:445288/1‑301`  
    `GCATGTTACTTCATGTTGTCAATACCTGTTTTTCGTGCGACTTATCAGGCTGTCTACTTATCCGGTTGACCCCGGTGTCGATTGGGATGGCTATTCGCCGTGTCCCTCTCGATGGCTGTAAGTATCCTATAGGTTAGACTTTAAGTCAATACTCTTTTTGATAAATTTTAAATTAATCTTTTAAGTCTCTTTAAGTTGTCTCTCCTTATAGTGAGTTGTATTAATAAAGACAATGATTAAAGGTTATCACTTTATGGCTAACAACAAGGCGTCTTTAGGTCTGGTCTTTATGTAGTGTCTT                                                                                                                                                                                                                                                                                                                               >  1:488629/1‑301`  
    `GCATGTTACTTCATGTTGTCAATACCTGTTTTTCGTGCGACTTATCAGGCTGTCTACTTATCCGGTTGACCCCGGTGTCGATTGGGATGGCTATTCGCCGTGTCCCTCTCGATGGCTGTAAGTATCCTATAGGTTAGACTTTAAGTCAATACTCTTTTTGATAAATTTTAAATTAATCTTTTAAGTCTCTTTAAGTTGTCTCTCCTTATAGTGAGTTGTATTAATAAAGACAATGATTAAAGGTTATCACTTTATGGCTAACAACAAGGCGTCTTTAGGTCTGGTCTTTATGTAGTGTCTT                                                                                                                                                                                                                                                                                                                               >  1:152681/1‑301`  
    `GCATGTTACTTCATGTTGTCAATACCTGTTTTTCGTGCGACTTATCAGGCTGTCTACTTATCCGGTTGACCCCGGTGTCGATTGGGATGGCTATTCGCCGTGTCCCTCTCGATGGCTGTAAGTATCCTATAGGTTAGACTTTAAGTCAATACTCTTTTTGATAAATTTTAAATTAATCTTTTAAGTCTCTTTAAGTTGTCTCTCCTTATAGTGAGTTGTATTAATAAAGACAATGATTAAAGGTTATCACTTTATGGCTAACAACAAGGCGTCTTTAGGTCTGGTCTTTATGTAGTGTCTT                                                                                                                                                                                                                                                                                                                               >  1:30466/1‑301`  
      `attTTACTTCATGTTGTCAATACCTGTGTTTCTTGCGACTTATCAGGCTGTCTACTTATCCGGTTGACACCGGTGTCGATTGGGATGGCTATTCGCCGTGTCCCTATCGATGGCTGTAAGTATCCTATAGGTTAGACTTTAAGTCAATACTCTTTTTGATAAATTTTAAATTAATCTTTTAAGTCTCTTTAAGTTGTCTCTCCTTATAGTGAGTTGTATTAATAAAGACAATGATTAAAGGTTATCACTTTATGGCTAACAACAAGGCGTCTTTAGGTCTGGTCTTTATGTAGTGTCTTTA                                                                                                                                                                                                                                                                                                                             <  1:225939/298‑1`  
      `ATGTTACTTCCATTTGTCCATACATTTTTTTCGTGCTACTTATCAGTCTGTCTACTTATCCGGTTGAGCCCGGTGTCGATTGGGATGGCTATTCGCCGTGTCCCTCTCGATTGCTGTAAGTATCCTATAGGTTAGACTTTAAGTCAATACTCTTTGTGCTAAATTTTAAATTAATCTTTTAAGTCTCTTTAAGTTGTCTCTCCTTATAGTGAGTTGTATTAATAAATACAATGATTAAAGGTTATCACTTTATGGCAAACAACAAGGCGTCTTTAGGTCTGGTCTTTATGTAGTCTCTTTA                                                                                                                                                                                                                                                                                                                             <  1:250902/301‑1`  
      `ATGTTACTTCATGTTGTCAATACCTGTTTTTCGTGCGACTTTTCAGGCTGTCTACTTATCCGGTTGACCCCTGTGTCGATTGGAGTGGCTATTCGCCGTGTCCCTCTCGATGGCTGTAAGTATCCTATAGGTTAGACTTTAAGTCAATACTCATTTTGATAAATTTTAAATTAATCTTTTAAGTCTCTTTAAGTTGTCTCTCCTTATAGTGAGTTGTATTAATAAAGACAATGATTAAAGGTTATCACTTTATGGCTAACAACAAGGCGTCTTTAGGTCTGGTCTTTATGTAGTGTCTTTA                                                                                                                                                                                                                                                                                                                             <  1:95126/301‑1`  
      `ATGTTACTTCATGTTGTCAATACCTGTTTTTCGTGCGACTTATCAGGCTGTCTACTTATCCGGTTGACCCCGGTGTCGATTGGGATGGCTATTCGCCGTGTCCCTCTCGATGGCTGTAAGTATCCTATAGGTTAGACTTTAAGTCAATACTCTTTTTGATAAATTTTAAATTAATCTTTTAAGTCTCTTTAAGTTGTCTCTCCTTATAGTGAGTTGTATTAATAAAGACAATGATTAAAGGTTATCACTTTATGGCTAAAAACAAGGCGTCTTTAGGTCTGGTCTTTGTGTAGTGTCTTTt                                                                                                                                                                                                                                                                                                                             >  1:345721/1‑300`  
      `ATGTTACTTCATGTTGTCAATACCTGTTTTTCGTGCGACTTATCAGGCTGTCTACTTATCCGGTTGACCCCGGTGTCGATTGGGATGGCTATTCGCCGTGTCCCTCTCGATGGCTGTAAGTATCCTATAGGTTAGACTTTAAGTAAATACTCTTTTCGATAAATTTTAAATTAATCTTTTAAGTCTCTTTAAGTTGTCTCTCCTTATAGTGAGTTGTATTAATAAAGACAATGATTAAAGGTTATCACTTTATGGCTAACAACAAGGCGTCTTTAGGTCTGGTCTTTATGTAGTGTCTTTA                                                                                                                                                                                                                                                                                                                             >  1:288627/1‑301`  
      `ATGTTACTTCATGTTGTCAATACCTGTTTTTCGTGCGACTTATCAGGCTGTCTACTTATCCGGTTGACCACGGTGTCGATTGGGATGGCTATTCGCCGTGTCCCTCTCGATGGCTGTAAGTATCCTATAGGTTAGACTTTAAGTCAATACTCTTTTTGATAAATTTTAAATTAATCTTTTAAGTCTCTTTAAGTTGTCTCTCCTTATAGTGAGTTGTATTAATAAAGACAATGATTAAAGGTTATCACTTTATGGCTAACAACAAGGCGTCTTTAGGTCTGGTCTTTATGTAGTGTCTTTt                                                                                                                                                                                                                                                                                                                             >  1:427837/1‑300`  
       `TGTTACTTCATGTTGTCAATACCTGTTTTTCGTGCGACTTATCAGGCTGTCTACTTATCCGGTTGACCCCGGTGTCGATTGGGATGGCTATTCGCCGTGTCCCTCTCGATGGCTGTAAGTATCCTATAGGTTAGACTTTAAGTCAATACTCTTTTTGATAAATTTTAAATTAATCTTTTAAGTCTCTTTAAGTTGTCTCTCCTTATAGTGAGTTGTATTAATAAAGACAATGATTAAAGGTTATCACTTTATGGCTAACAACAAGGCGTCTTTAGGTCTGGTCTTTATGTGGTGTCTTTAG                                                                                                                                                                                                                                                                                                                            >  1:372099/1‑301`  
       `TGTTACTTCATGTTGTCAATACCTGTTTTTCGTGCGACTTATCAGGCTGTCTACTTATCAGGTTGACCCCGGTGTCGATTGGTATGGCTATTCGCCGTTTCCCTCTCGATGGCTGTAAGTATCCTATAGGTTAGACTTTAAGTCAATACTCTTTTTGATAAATTTTAAATTAATCTTTTAAGTCTCTTTAGGTTGTCTCTCCTTATCTTGAGTTGTATTAATCAAGACAATGATTAAAGTTTATCACTTTTTGTCTAACAACAAGGCGTCTTTAGGTCTGTTCTTTATGTAGTGTCTTTAG                                                                                                                                                                                                                                                                                                                            >  1:164791/1‑301`  
        `ttttCTTCCTGTTTTCAAGACCTGTGTTTAGGGCAACGTCTCAGTCTGGTGACTTATCCGGTTGATCTAGGTGTGGATTGGGATGGCTATGCGCCGTGCCCCTCTCTATGGCTGTAAGTATCCTATTGGTTAGACTTTAAGTCAATATTCTTTTTGATAAATTTTAAATTAATCTTTTAAGTCTCTTTAAGTTGTCTATCCTTATAGTGAGTTGTATTAATAAAGACAATGATTAAAGGTTATCACTTTATGGCTAACAACAAGGCGTCTTTAGGTCTGGTCTTTATGTAGTGTCTTTAGG                                                                                                                                                                                                                                                                                                                           <  1:315738/297‑1`  
        `tTTACTTCATGTTGTCAATACCTGTTTTTCGTGCGACTTATCAGGCTGTCTACTTATCCGGTTGACCCCGGTGTCGATTGGGATGGCTATTCGCCGTGTCCCTCTCGATGGCTGTAAGTATCCTATAGGTTAGACTTTAAGTCAATACTCTTTTTGATAAATTTTAAATTAATCTTTTAAGTCTCTTTAAGTTGTCTCTCCTTATAGTGAGTTGTATTAATAAAGACAATGATTAAAGGTTATCACTTTATGTCTAACAACAAGGCGTCTTTAGGTCTGGTCTTTATGTAGTGTATTTAGG                                                                                                                                                                                                                                                                                                                           <  1:60094/300‑1`  
        `tTTACTTCATGTTGTCAATACCTGTTTTTCGTGCGACTTATCAGGCTGTCTACTTATCCGGTTGACCCCGGTGTCGATTGGGATGGCTATTCGCCGTGTCCCTCTCGATGGCTGTAAGTATCCTATAGGTTAGACTTTAAGTCAATACTCTTTTTGATAAATTTTAAATTAATCTTTTAAGTCTCTTTAAGTTGTCTCTCCTTATAGTGAGTTGTATTAATAAAGACAATGATTAAAGGTTATCACTTTATGGCTAACAACAAGGCGTCTTTAGGTCTGGTCTTTATGTAGTGTCTTTAGG                                                                                                                                                                                                                                                                                                                           <  1:245974/300‑1`  
        `aagACTTCATGTTGTCAATACCTGTTTTTCGTGCTACTTATCAGGCTGTCTACTTATCCGGTTGACCCCGGTGTCGATTGGGATGGCTATTCGCCGTGTCCCTCTCGATGGCTGTAAGTATCCTATAGGTTAGACTTCAAGTCAATACTCTTTTTGATAAATTTTAAATTAATCTTTTAAGTCTCTTTAAGTTGTCTCTCCTTATAGTGAGTTGTATTAATAAAGACAATGATTAAAGGTTATCACTTTATGGCTAACAACAAGGCGTCTTTAGGTCTGGTCTTTATGTAGTGTCTTTAGG                                                                                                                                                                                                                                                                                                                           <  1:480168/298‑1`  
        `GTTACTTCATGTTTTATATACCTGTTTTTCGTGCGACTTATCAGGCTGTATACTTATCCGGTTGACCCCGGTGTCGATTGGGATGGCTATTCGCCGTGTCCCTCTCGATGGCTGTAAGTATACTATAGGTTAGACTTTAAGGCAATACTGTTTTTGATAAATGTTAAATTAATCTTTTAAGTCTCTTGAAGTTGTCTCTCCTTATAGTGAGTTGTATTAATAAAGACAATGATTAAAGGTTATCACGTTATGGCTAACAACAAGGCGTCTTTAGGTCTGGTCTTTATGTAGTGTCTTTAGG                                                                                                                                                                                                                                                                                                                           <  1:217485/301‑1`  
        `GTTACTTCATGTTGTCAATACCTGTTTTTCGGGCGACTTATCAGGCTGTCTACTTATCCGGTTGACCCCGGTGTCGATTGTGATGGCTATTCGCCGTGTCCCTCTCGATGGCTGTAAGTATCCTATAGGTTAGACTTTAAGTCAATACTCTTTTTGATAAATTTTAAATTAATCTTTTAAGTCTCTTTAAGTTGTCTCTCCTTATAGTGAGTTGTATTAATAAAGACAATGATTAAAGGTTATCACTTTATGGCTAACAACAAGGCGTCTTTAGGTCTGGTCTTTATGTAGTGTCTTTAGG                                                                                                                                                                                                                                                                                                                           <  1:129345/301‑1`  
         `taatagagaacgGTCAATTCCTGTTTTTCGTTCGACTGTGTAGGCTGTTTGCTTATCCGGTTGACCCCGGTGTCGTTTGGTATTGCTTTTCGCCGTGTCCCTCTCGATGGCTGTAAGTATCCTTTAGGTTAGACTTTAAGTCAATACTCTTTTTGATAAATTTTAAATTAATCTTTTAAGTCTCTTTAAGTTGTCTCTCCTTATAGTGAGTTGTATTAATAAAGACTATGATTAAAGGTTATCACTTTATGGCTAACAACAAGGCGTCTTTAGGTCTGGTCTTTATGTAGTGTCTTTAGGT                                                                                                                                                                                                                                                                                                                          <  1:497583/289‑1`  
          `tagTTCAAGTTGTCAATACCTGTTTAGCTAGCGAATTACTAGGCTGTCTACTTATCCGTTTGACCCCGGTGTCGATTGGTATGGCTATTCGCCTGGTCCCTACCGATGTCTGTAAGTATCCTAGAGTTTATACTTGAAGTCAATACTCTTTTTGATAAATTTTAAATTAATCTTTTAAGTCTCTTTAAGTTGTCTCTCCTTATAGTGAGTTGTATTAATAAAGACAATGATTAAAGGTTATCACTTTATGGCTAACAACAAGGCGTCTTTAGGTCTGGTCTTTATGTAGTGTCTTTAGGTC                                                                                                                                                                                                                                                                                                                         <  1:333141/298‑1`  
          `gACTTCATGTTGTCAATACCTGTTTTTCGTGCGACTTATCAGGATGTCTACTTATACGGTTGACCCCGGTGTCGATTGGGATGGCTATTCGCCGTGTCCCTCTCGATGGCTGTAAGTATCCTATAGGTTAGACTTTAAGTCAATACTCTTTTTGATAAATTTTAAATTAATCTTTTAAGTCTCTGTAAGTTGTCTCTCCTTATAGTGAGTTGTATTAATAAAGACAATGATTAAAGGTTATCACTTTATGGCTAACAACAAGGCGTCTTTAGGTCTGGTCTTTATGTAGTGTCTTTAGGTC                                                                                                                                                                                                                                                                                                                         <  1:470447/300‑1`  
          `ataagagacagGTCAATACCTGTTTTTCGTGCGACTTATCAGGCTGTCTACTTATCCGGTTGACCCCGGTGTCGATTGTGATGGCTATTCGCCGTGTCCCTCTCGATGGCTGTAAGTATCCTATAGGTTAGACTTTAAGTCAATACTCTTTTTGATAAATTTTAAATTAATCTTTTAAGTCTCTTTAAGTTGTCTCTCCTTATAGTGAGTTGTATTAATAAAGACAATGATTAAAGGTTATCACTTTATGGCTAACAACAAGGCGTCTTTAGGTCTGGTCTTTATGTAGTGTCTTTAGGTC                                                                                                                                                                                                                                                                                                                         <  1:337594/290‑1`  
          `ataagagacagGTCAATACCTGTTTTTCGTGCGACTTATCAGGCTGTCTACTTATCCGGTTGACCCCGGTGTCGATTGGGATGGCTATTCGCCGTGTCCCTCTCGATGGCTGTAAGTATCCTATAGGTTAGACTTTAAGTCAATACTCTTTTTGATAAATTTTAAATTAATCTTTTAAGTCTATTTAAGTTGTCTCTCCTTATAGTGAGTTGTATTAATAAAGACAATGATTAAAGGTTATCACTTTATGGCTAACAACAAGGCGTCTTTAGGTCTGGTCTTTATGTAGTGTCTTTAGGTC                                                                                                                                                                                                                                                                                                                         <  1:269425/290‑1`  
          `aaataagagacagCAATACCTGTTTTTCGTGCGACTTATCAGGCTGTCTACTTATCCGGTTGACCACGGTGTCGATTGGGATGGCTATTCGCCGTGTCCCTCTCGATGGCTGTAAGTATCCTGTAGGTTAGACTTTAAGTCAATACTCTTTTTGATAAATTTTAAATTAATCTTTTAAGTCGCTTTAAGTTGTCTCTCCTTATAGTGAGTTGTATTAATAAAGACAATGATTAAAGGTTATCACTTTATGGCTAACAACAAGGCGTCTTTAGGTCTGGTCTTTATGTAGTGTCTTTAGGTC                                                                                                                                                                                                                                                                                                                         <  1:348756/288‑1`  
          `TACTTCTTGTTGTCAATACCTGTTTTTCGTGCGACTTATCAGGCTGTCTACTTATCCGGTTGACCCCGGTGTCGATTGGGATGGCTATTCGCCGTGTCCCTCTCGATGGCTGTAAGTATCCTATAGGTTAGACTTTAAGTAAATACTCTTTTTGATAAATTTTAAATTAATCTTTTAAGTCTCTTTAAGTTGTCACTCCTTATAGTGAGTTGTATTAATAAAGACAATGATTAAAGGTTATCACTTTATGGCTAACAACAAGGCGTCTTTAGGTCTGGTCTTTATGTAGTGTCTTTAGGTC                                                                                                                                                                                                                                                                                                                         <  1:294839/301‑1`  
          `TACTTCATGTTGTCAATACCTGTTTTTGGTGCGACTTATCAGGCTGTCTACTTATCTGGTTGACCCCGGTGTCGATTGGGATGGCTATTCGCCGTGTCCCTCTCGATGGCTGTAAGTATCCTATAGGTTAGACTTTAAGTCAATACTCTTTTTGATAAATTTTAAATTAATCTTTTAAGTCTCTTTAAGTTGTCTCTCCTTATAGTGAGTTGTATTAATAAAGACAATGATTAAAGGTTATCACTTTATGGCTAACAACAAGGCGTCTTTAGGTCTGGTCTTTATGTAGTGTCTTTAGGTC                                                                                                                                                                                                                                                                                                                         <  1:267240/301‑1`  
          `TACTTCATGTTGTCAATACCTGTTTTTCGTGCGACTTATCAGGCTTTCTACTTATCCGGTTGACCCCGGTGTCGATTGGGATGGCTATTCGCCGTGTCCCTCTCGATGGCTGTAAGTATCCTATAGGTTAGACTTTAAGTCAATACTCTTTTTGATAAATTTTAAATTAATCTTTTAAGTCTCTTTAAGTTGTCTCTCCTTATAGTGAGTTGTATTAATAAAGACAATGATTAAAGGTTATCACTTTATGGCTAACAACAAGGCGTCTTTAGGTCTGGTCTTTATGTAGTGTCTTTAGGTC                                                                                                                                                                                                                                                                                                                         <  1:318649/301‑1`  
          `TACTTCATGTTGTCAATACCTGTTTTTCGTGCGACTTATCAGGCTGTCTACTTATCCGGTTGACCCCGGTGTCGATTGGGATGGCTATTCGCCGTGTCCCTCTCGATGGCTGTAAGTATCCTATAGGTTAGACTTTAAGTCAATACTCTTTTTGATAAATTTTAAATTAATCTTTTAAGTCTCTTTAAGTTGTCTCTCCTTATAGTGAGTTGTATTAATAAAGACAATGATTAAAGGTTATCACTTTATGGCTAACAACAAGGCGTCTTTAGGTCTGGTCTTTATGTAGTGTCTTTAGGTC                                                                                                                                                                                                                                                                                                                         <  1:461123/301‑1`  
          `TACTTCATGTTGTCAATACCTGTTTTTCGTGCGACTTATCAGGCTGTCTACTTATCCGGTTGACCCCGGTGTCGATTGGGATGGCTATTCGCCGTGTCCCTCTCGATGGCTGTAAGTATCCTATAGGTTAGACTTTAAGTCAATACTCTTTTTGATAAATTTTAAATTAATCTTTTAAGTCTCTTTAAGTTGTCTCTCCTTATAGTGAGTTGTATTAATAAAGACAATGAATAAAGGTTATCACTTTATGGCTAACAACAAGGCGTCTTTAGGTCTGGTCTTTATGTAGTGTCTTTAGGTC                                                                                                                                                                                                                                                                                                                         <  1:212795/301‑1`  
          `TACTTCATGTTGTCAATACCTGTTTTTCGGGCGACTTATCAGGCTGTCTACTTATCCGGTTGACCCCGGTGTCGATTGGGATGGCTATTCGCCGTGTTCCTCTCGATGGCTGAAAGTATCCTATAGGTTAGACTTTAAGTCAATACTCTTTTTGATAAATTTTAAATTAATCTTTTAAGTCTCTTTAAGTTGTCTCTCCTTATAGTGAGTTGTATTAATAAAGACAATGATTAAAGGTTATCACTTTATGGCTAACAACAAGGCGTCTTTAGGTCTGGTCTTTATGTAGTGTCTTTAGGTC                                                                                                                                                                                                                                                                                                                         <  1:30835/301‑1`  
          `TACTTCATGTGGTCAATACCTGTGTTTCGTGCGACTTATCAGGCTGTCTACTTATCGGGTTGACCCCGGTGTCGATGGGGATGGCTATTCGCCGTGTCCCTATCGATGGCGGTAAGTAGCCTATAGGTTAGACTTTAAGTCAATACTCTTTTTGATAAATTTTAAATTAATCTTTTAAGTCTCTTTAAGTTGTCTCTCCTTATAGTGAGATGTATGAATAAAGACAATGATTAAAGGTTATCACTTTATGGCTAACAACAAGGCGTCTTTAGGTCTGGTCTTTATGTAGTGTCTTTAGGTC                                                                                                                                                                                                                                                                                                                         <  1:5555/301‑1`  
          `TACTTCAGGTTGTCAATACCTGTTTTTCGTGCTACTGATCAGGCGGGTTAATTATCCGGTTGACCCCGGTGTCGATTGGGTTGGCTATTAGCCGTGTCCCTCTCGATGGCTGTAAGTATCCTATATGTTAGACTTTAAGTCAATATTCTTTTTGATAAATTTTAAATTAATCTTTTAAGTCTCTTTAAGTTGTCTCTCCTTATAGTGAGTTGTATTAATAAAGACAATGATTAAAGGTTATCACTTTATGGCTAACAACAAGGCGTCTTTAGGTCTGGTCTTTATGTAGTGTCTTTAGGTC                                                                                                                                                                                                                                                                                                                         <  1:291395/301‑1`  
           `ACTTCATGTTGTCAATACCTGTTTTTCGTGCGACTTATCAGGCTGTCTACTTATCCGGTTGACCCCGGTGTCGATTTGGATGGCTATTCGCCGTGTCCCTCTCGATGGCTGTAAGTATCCTATAGGTTAGACTTTAAGTCAATACTCTTTTTGATAAATTTTAAATTAATCTTTTAAGTCTCTTTAAGTTGTCTCTCCTTATAGTGAGTTGTATTAATAAAGACAATGATTAAAGGTTATCACTTTATGGCTAACAACAAGGAGTCTTTAGGTCTGGTCTTTTTGTAGTGTCTTTAGGTCc                                                                                                                                                                                                                                                                                                                        >  1:88583/1‑300`  
           `ACTTCATGTTGTCAATACCTGTTTTTCGTGCGACTTATCAGGCTGTCTACTTATCCGGTTGACCCCGGTGTCGATTGGGATGGCTATTCGCCGTGTCCCTCTCGATGGCTGTAAGTATCCTATAGGTTAGACTTTAAGTCAATACTCTTTTTGATAAATTTTAAATTAATCTTTTAAGTCTCTTTAAGTTGTCTCTCCTTATAGTGAGTTGTATTAATAAAGACAATGATTAAATGTTATCACTTTATGGCTAACAACAAGGCGTCTTTAGGTCTGGTCTTTATGTTGTGTCTTTAGGTCT                                                                                                                                                                                                                                                                                                                        >  1:396009/1‑301`  
           `ACTTCATGTTGTCAATACCTGTTTTTCGTGCGACTTATCAGGCTGTCTACTTATCCGGTTGACCCCGGTGTCGATTGGGATGGCTATTCGCCGTGTCCCTCTCGATGGCTGTAAGTATCCTATAGGTTAGACTTTAAGTCAATACTCTTTTTGATAAATTTTAAATTAATCTTTTAAGTCTCTTTAAGTTGTCTCTCCTTATAGTGAGTTGTATTAATAAAGACAATGATTAAATGTTATCACTTTATGGCTAACAACAAGGCGTCTTTAGGTCTGGTCTTTATGTAGTGTCTTTAGGTCT                                                                                                                                                                                                                                                                                                                        >  1:105160/1‑301`  
           `ACTTCATGTTGTCAATACCTGTTTTTCGTGCGACTTATCAGGCTGTCTACTTATCCGGTTGACCCCGGTGTCGATTGGGATGGCTATTCGCCGTGTCCCTCTCGATGGCTGTAAGTATCCTATAGGTTAGACTTTAAGTCAATACTCTTTTTGATAAATTTTAAATTAATCTTTTAAGTCTCTTTAAGTTGTCTCTCCTTATAGTGAGTTGTATTAATAAAGACAATGATTAAAGGTTATCACTTTATGGCTAACAACAAGGCGTCTTTAGGTCTGGTCTTTATGTAGTGTCTTTAGGTCT                                                                                                                                                                                                                                                                                                                        >  1:495524/1‑301`  
           `ACTTCATGTTGTCAATACCTGTTTTTCGTGCGACTTATCAGGCTGTCTACTTATCCGGTTGACCCCGGTGTCGATTGGGATGGCTATTCGCCGTGTCCCTCTCGATGGCTGTAAGTATCCTATAGGTTAGACTTTAAGTCAATACTCTTTTTGATAAATTTTAAATTAATCTTTTAAGTCTCTTTAAGTTGTCTCTCCTTATAGTGAGTTGTATTAATAAAGACAATGATTAAAGGTTATCACTTTATGGCTAACAACAAGGCGTCTTTAGGTCTGGTCTTTATGTAGTGTCTTTAGGTCT                                                                                                                                                                                                                                                                                                                        >  1:280505/1‑301`  
           `ACTTCATGTTGTCAATACCTGTTTTTCGTGCGACTTATCAGGCTGTCTACTTATCCGGTTGACCCCGGTGTCGATTGGGATGGATATTCGCCGTGTCCCTCTCGATGGCTGTAAGTATCCTATAGGTTAGACTTTAAGTCAATACTCTTTTTGATAAATTTTAAATTAATCTTTTAAGTCTCTTTAAGTTGTCTCTCCTTATAGTGAGTTGTATTAATAAAGACAATGATTAAAGGTTATCACTTTATGGCTAACAACAAGGCGTCTTTAGGTCTGGTCTTTATGTAGTGTCTTTAGGTCT                                                                                                                                                                                                                                                                                                                        >  1:450692/1‑301`  
            `CTTCATGTTGTCAATACCTGTTTTTCGTGCGACTTATCAGGCTGTCTACTTATCCGGTTGACCCCGGTGTCGATTGGGATGGCTATTCGCCGTGTCCCTCTCGATGGCTGTAAGTATCCTATAGGTTAGACTTTAAGTCAATACTCTTTTTGATAAATTTTAAATTAATCTTTTAAGTCTCTTTAAGTTGTCTCTCCTTATAGTGAGTTGTATTAATAAAGACAATGATTAAAGGTTATCACTTTATGGCTAACAACAAGGCGTCTTTAGGTCTGGTCTTTATGTAGTTTCTTTAGGctgt                                                                                                                                                                                                                                                                                                                       >  1:276866/1‑297`  
              `TCATGTTGTCAATACCTGTTTTTCGTGCGACTTATCAGGCTGTCTACTTATCCGGTTGACCCCGGTGTCGATTGGGATGGCTATTCGCCGTGTCCCTCTCGATGGCTGTAAGTATCCTATAGGTTAGACTTTAAGTCAATACTCTTTTTGATAAATTTTAAATCAATCTTTTAAGTCTCTTTAAGTTGTCTCTCCTTATAGTGAGTTGTATTAATAAAGACAATGATTAAAGGTTATCACTTTATGGC‑AACAACAAGGCGTCTTTAGGTCTGGTCTTTATGTAGTGTCTTTAGGTCTGGTC                                                                                                                                                                                                                                                                                                                    <  1:238007/301‑1`  
                `ggcagtTCAATGCCTGTTTTTCGTGCGGCTTATCAGGCTTTCTACTTATCCGGTTGACCCCGGTGTCGATTGGGATTGCTATTCGCCGTGTCCCTTTCGATGGCTGTAAGTATCCTATAGGTTAGCCTTTAAGTCAATACTCTTTTTGATAAATTTTAAATTAATCTTTTAAGTCTCTTTAAGTTGTCTCTCCTTATAGTGAGTTGTATTAATAAAGACAATGATTAAAGGTTATCACTTTATGGCTAACAACAAGGCGTCTTTAGGTCTGGTCTTTATGTAGTGTCTTTAGGTATGGTCT                                                                                                                                                                                                                                                                                                                   <  1:31984/295‑1`  
                `ATGTTGTCAATACCTGTTTTTCGTGCGACTTATCAGGCTGTCTACTTATCCGGTTGACCCCGGTGTCGATTGGGATGGCTATTCGCCGTGTCCCTCTCGATGGCTGTAAGTATCCTATAGGTTAGACTTTAAGTCAATACTCTTTTTGATAAATTTTAAATTAATCTTTTAAGTCTCTTTAAGTTGTCTCTCCTTATAGTGAGTTGTATTAATAAAGACAATGATTAAAGGTTATCACTTTATGGCTAACAACAAGGCGTCTTTAGGTATGGTCTTTATGTAGTGTCTTTAGGTactgtcc                                                                                                                                                                                                                                                                                                                   >  1:361589/1‑294`  
                    `TGTCAATACCTGTTTTTCGTGCGACTTATCAGGCTGTCTACTTATCCGGTTGACCCCGGTGTAGATTGGGATGGCTATTCGCCGTGTCCCTCTCGATGGCTGTAAGTATCCTATAGGTTAGACTTTAAGTCAATACTCTTTTTGATAAATTTTAAATTAATCTTTTAAGTCTCTTTAAGTTGTCTCTCCTTATAGTGAGTTGTATTAATAAAGACAATGATTAAAGGTTATCACTTTATGGCTAACAACAAGGCGTCTTTAGGTCTGGTCTTTATGTAGTGTCTTTTGGTCTGGTCctgtt                                                                                                                                                                                                                                                                                                               >  1:401502/1‑296`  
                            `CCTGTTTTTCGTGCGACTTATCAGGCTGTCTACTTATCCGGTTGACCCCGGTGTCGATTGGGATGGCTATTCGCCGTGTCCCTCTCGATGGCTGTAAGTATCCTATAGGTTAGACTTTAAGTCAATACTCTTTTTGATAAATTTTAAATTAATCTTTTAAGTCTCTTTAAGTTGTCTCTCCTTATAGTGAGTTGTATTAATAAAGACAATGATTAAAGGTTATCACTTTATGGCTAACAACAAGGCGTCTTTAGGTCTGGTCTTTATGTAGTGTCTTTActgtctcttatacacatctccg                                                                                                                                                                                                                                                                                                       >  1:114825/1‑279`  
                                           `ACTTATCAGGCTGTCTACTTATCCGGTTGACCCCGGTGTCGATTGTGATGGCTATTCGCCGTGTCCCTCTCGATGGCTGTAAGTATCCTATAGGTTAGACTTTAAGTCAATACTCTTTTTGATAAATTTTAAATTAATCTTTTAAGTCTCTTTAAGTTGTCTCTCCTTATAGTGAGTTGTATTAATAAAGACAATGATTAAAGTTTATCACTTTATGGCTAACAACAAGGCGTCTTTAGGTCTGGTCTTTATGTAGTGTCTTTAGTTCCTGTCTcttatacacatctccgagcccacgaga                                                                                                                                                                                                                                                                                        >  1:64311/1‑274`  
                                                                                                                                            `GACTTTAAGTCAATACTCTTTTTGATAAATTTTAAATTAATCTTTTAAGTCTCTTTATGTTGTCTCTCCTTATAGTGAGTTGTATTAATAAAGCTAATGATTAAAGGTTATCACTTTATAGCTAACAACAAAGCGTCTTTAGGTATGGTCTTTCTGTAGTGTATTTACGTCGGGTCTTTATGTTTAAACTTTAAGATTGGCGTTGACTTAATGGGTCTTTAGTTGTAGGCTTTAGGTGTTGGCTTTAGTCTGGACGTTAGGAGGTACCTTTAGTAGGATACTTTATGAGAGTGTAACAGAT                                                                                                                                                                                       >  1:503758/1‑301`  
                                                                                                                                                                                                                                                                                             `tgtataagagacagggttgTTAGGGCTGGTCGTTATGTTGAAACTTGAAGATAGGCGTTGACTTGATGGGTCTTTAGGTGTAGGCGTTAGGTGTTGGCTTTAGGATGGACGTTAGGAGGTGACTTTAGGAGGATACTTTAGGAGACTGTAACAGATAGGGACACAGAGAGACACTCAAGGTAACACCCAAAGACAAACCCAAGGGTCATCCCCAACCCTTAGGGAACCCTCAAGGTCAACCGAAGGTTGACTTTAGGTGATTGGCTGGGCTTTAGGTA‑CCCCTATGGGGGAACTTTAGGTC                                     <  1:369330/282‑1`  
                                                                                                                                                                                                                                                                                             `ggtataggagaccggggtTTTAGGCCGGGGTTTTATGTTTAAACTTTAAGATAGGCGTTGACTTGATGGGTCTTTAGTTGTAGGATTTAGGTGTTGGCCTTAGGGTGGACGTTAGGAGGTGACTTTAGGAGGATACTTTAGGAGACTGTAACAGATAGGGACACAGAGAGACACTAAAGGTAACACCCAAAGACAAACCCAAGGGTCATCCCCAACCCTTAGGGAACCCTCAAGGTCAACCGAAGGTTGACTTTAGGTGATTGGCTGGGCTTTAGGTA‑CCCCTATGGGGGAACTTTAGGTt                                     <  1:92781/283‑2`  
                                                                                                                                                                                                                                                                                             `atgtgtataagagacagCTTTAGGTTTGGTCTTTATGTTTAAACTTTAAGATAGGCTTTGACTTGATGGGTCTTTAGGTGTAGGCTTTAGGTGTTGGCTTTAGGATGGACGTTAGGAGGTGACTTTAGGAGGATACTTTAGGAGACTGTAACAGATAGGGACACAGAGAGACACTCAATGTAACACCCAAAGACAAACCCAAGGGTCATCCCCAACCCTTAGGGAACCCTCAAGGTCAACCGAAGGTTGACTTTAGGTGATTGGCTGGGCTTTAGGTA‑CCCCTATGGGGGAACTTTAGGgc                                     <  1:257156/284‑3`  
                                                                                                                                                                                                                                                                                             `atgtgtataagagacagCTTTAGGTCTGGTTTTTATGTTTAAACTTTAAGATAGGAGTTGACTTGATGGGTCTTTAGGTGTAGGCTTTAGGTGTTGGCTTTAGGATGGACGTTAGGAGGTGACTTTAGTAGGATACTTTAGGAGACTGTAACAGATAGGGACACAGAGAGACACTCAAGGTAACACCCAAAGACAAACCCAAGGGTCATCCCCAACCCTTAGGGAACCCTCAAGGTCAACCGAAGGTTGACTTTAGGTGATTGGCTGGGCTTTAGGTA‑CCCCTATGGGGGAACTTTAGGTC                                     <  1:149691/284‑1`  
                                                                                                                                                                                                                                                                                             `aaagagcaaggtttTGTCTTTAGGTTTTGTCTTTAAGTTTAAACTTTAAGGTAGGCGTTGAATTGAAGGGTTTTTAGGTTTAGGCTTTAGGATTTTGCTTTGAGATGGACGGTAGGAGGTGACTTTAGGAAGATACTTTAGGAGTCTGTTACAGATAGGGACACAGAGAGACACTAAAGGTAACACCCAAAGACAAACCCAAGGGTCATCCCCAACCCTTAGGGAACCCTCAAGGTCAACCGAAGGTTGACTTTAGGTGATTGGCTGGGCTTTAGGTA‑CCCCTATGGGGGAACTTTAGGTC                                     <  1:414824/287‑1`  
                                                                                                                                                                                                                                                                                                  `tttagtgttTGTCTTTATTTCTGGTCTTTATGTTTAAACTTTAAGATAGGCGTTGACTTGATGGGTCTTTAGGTGTATGCTTTAGTTGTTGGCTTCAGGATGGACGTTAGGAGGTGACTTTAGGAGGATACTTTAGGAGACTGTAACAGATAGGGACACAGATAGACACTCAAGGTAACACCCAAAGACAAACCCAAGGGTCATCCCCAACCCTTAGGGAACCCTCAAGGTCAACCGAAGGTTGACTTTAGGTAATTGGCTGGGCTTTAGCTA‑CCCCTATGGGGGAACTTTAGGTCCGTAC                                <  1:282150/292‑1`  
                                                                                                                                                                                                                                                                                                  `ttaggaaatttgtataagagactGTCTTTATTTTTAACCTTTTAAATAGGCGTTTACTTCATGCGTCTTTAGGTGTAGGATTTAGTTTTTGGCTTTAGGTTGGACGTTAGGAGGTGACTTTAGGAGGATACTTTAGGGGACTGTAACAGATAGGGACACAGAGAGACACTCAAGGTAACACCCAAAGACAAACCCAAGTGTCATCCCCAACCCTTAAGGAACCCTCAAGTTCAACCGAAGGTTGACTTTAGGTGATTGGCTGGGCTTTAGGTA‑CCCCTATGGGGGAACTTTAGGTCCGTAC                                <  1:158703/278‑1`  
                                                                                                                                                                                                                                                                                                  `tataagagacagcgTTAGGTCGGGTCTTTATGTTTAAACTTTAAGATAGGCGTTGACTTGATGGGTCTTTAGGTGTAGGCTGTAGGTGTTGGCTTTAGGATGGACGTTAGGAGGTGACTTTAGGAGGATACTTTAGGAGACTGTAACAGATAGGGACACAGAGAGACACTCAAGGTAACACCCAAAGACAAACCCAAGGGTCATCCCCAACCCTTAGGGAACCCTCAAGGTCAACCGAAGGTTGACTTTAGGTGATTGGCTGGGCTTTAGGTA‑CCCCTATGGGGGAACTTTAGGTCCGTAC                                <  1:193968/287‑1`  
                                                                                                                                                                                                                                                                                                  `tataagagaaagCTTTAGGTCTGGTCTTTATGTTTAAACTTTAAGATAGGCGTTTACTTGATGGGTCTGTAGGTGTAGGCTTTAGGTGTTTGCTGTAGGATGTACGTTAGGAGGTGACTTTAGGAGGATACTTTAGGAGACTGTAACAGATAGGGACACAGAGAGACACTCAAGGTAACACCCAAAGACAAACCCAAGGGTCATCCCCAACCCTTAGGGAACCCTCAAGGTCAACCGAAGGTTGACTTTAGGTTATTGGCTGGGCTTTAGGTA‑CCCCTATGGGGGAACTTTAGGTCCGTAC                                <  1:439557/289‑1`  
                                                                                                                                                                                                                                                                                                  `cggagatgtgtataagagacagGGTCTTTATGTTTAAACTTTAAGATAGGCGTTGACTTGATGGGTCTTTAGGTGTAGGCTTTAGGTGTTGGCTTTAGGATGGACGTTAGGAGGTGACTTTAGGATGATACTTTAGGAGACTGTAACAGATAGGGACACAGAGAGACACTCAAGGTAACACCCAAAGACAAACCCAAGGGTCATCCCCAACCCTTAGGGAACCCTCAAGGTCAACCGAAGGTTGACTTTAGGTGATTGGCTGGGCTTTAGGTA‑CCCCTATGGGGGAACTTTAGGTCCGTAC                                <  1:18162/279‑1`  
                                                                                                                                                                                                                                                                                                    `tagttttgggCTTTAGGGCTGGTCTTTATGTTTAAACTTTAAGATAGGTGTTGACTGGATGGGTTGTGAGGTGTAGGATTTAGGTGTTGGGTTTAGGATGGACGTTAGGAGGTGACTTTAGGAGGATACTTTAGGATACTGTAACAGATAGGGACACAGAGAGACACTCAAGGTAACACCCAAAGACAAACCCAAGGGTCATCCCCAACCCTTAGGGAACCCTCAAGGTCAACCGAAGGTTGACTTTAGGTGATTGGCTGGGCTTTAGGTA‑CCCCTATGGGGGAACTTTAGGTCCGTACAC                              <  1:465363/291‑1`  
                                                                                                                                                                                                                                                                                                    `taggtttTGTCTTTATTTCTTGTCTTTTTGTTTTAACTTTAAGATTGTCGTTGACTTTATGGGTCTTTAGGTGTAGGCTTTAGGTGTTTGCTTTAGGATGGACCTTAGGATGTGACTTTAGGAGGATACTTTAGGAGACTGTAACAGATAGGGACACAGAGAGACACTCAAGGTAACACCCAAAGACAAACCCAAGGGTCATCCCCAACCCTTAGGGAACCCTCAAGGTCAACCGAAGGTTGACTTTAGGTGATTTGCTGGGCTTTAGGTA‑CCCCTATGGGGGAACTTTAGGTCCGTACAC                              <  1:383606/294‑1`  
                                                                                                                                                                                                                                                                                                    `taggtgtgggtTTTAGGTCTGGTCTTTATGTTTAAACTTTAAGATAGGCGTTTACTTGATGGGTCTTTAGGTGTAGGCTTTAGGTGTTGGCTTTAGGATGGACGTTAGGAGGTTACCTTAGGAGGATACTTTAGGAGACTGTAACAGAGAGGGACAAAGGGAGACACTCAAGGTAACACCCAAAGACAAACCCAAGGGTCATCCCCAACCCTTAGGGAACCCTCAAGGTCAACCGAAGGTTGACTTTAGGGGATTGGCTGGGCTTTAGGTA‑CCCCTATGGGGGAACTTTAGGTCCGTACAC                              <  1:386241/290‑1`  
                                                                                                                                                                                                                                                                                                    `taggtatgggcgTTAGGTCTGGTCTTTATGTTTAAACTTTAAGATAGGCGGTGAATTGATGGGTCTTTAGGTGTAGGCTTTAGGTGTTGGCTTTAGGATGGACGTTAGGAGGTGAATTTAGGAGGATACTTTAGGAGACTGTAACAGATAGGGAAAAAGAGAGACACTCAAGGTAACACCCAAAGACAAACCCAAGGGTAATCCCCAACCCTTAGGGAACCCTCAAGGTCAACCGAAGGTTGACTTTAGGTGATTGGCTGGGCTTTAGGTA‑CCCCTATGGGGGAACTTTAGGTCCGTACAC                              <  1:144697/289‑1`  
                                                                                                                                                                                                                                                                                                    `tagggatgGTCTTTAGGTCTGGTCTTTATGTTTAAACTTTAAGATAGGCGTTGGCTTGATGGGTCTTTAGGGGTAGGCTTTAGGTGTTGGCTTTAGGATGGACGTTATGAGGTGACTTTATGAGGATAATTTAGGAGACTGTAAAAGATAGGGAAAGAGAGAGACACTCAAGGTAACACCCAAAGACAAACCCAAGGGTCATCCCCAACACTTAGGGAACCCTAAAGGTCAACCGAAGGTTGACTTTAGGTGATTGGCTGGGCTTTAGGTA‑CCCCTATGGGGGAACTTTAGGTCCGTACAC                              <  1:261264/293‑1`  
                                                                                                                                                                                                                                                                                                    `taagagacagCTTTAGGTCTGGTCTTTATGTTTAAACTTTAAGATAGGCGTTGACTTGATGGGTCTTTAGGTGTAGGCTTTAGGTGTTGGCTTTAGGATGGACGTTAGGAGGTGACTTTAGGAGGATACTTTAGGAGACTGTAACAGATAGGGACACAGAGAGACACTAAAGGTAACACCCAAAGACAAACCCAAGGGTCATCCCCAACCCTTAGGGAACCCTCAAGGTCAACCGAAGGTTGACTTTAGGTGATTGGCTGGGCTTTAGGTA‑ACCCTATGGGGGAACTTTAGGTCCGTACAC                              <  1:13348/291‑1`  
                                                                                                                                                                                                                                                                                                    `gtataagggacattgtGTCTTGTCTTTATGTTTAAACTTTAAGAATGGCGTTGAATTGATGGATCTTTAGGTGTTAGATTTAGGTGTTGGCTTTAGGATGGACCTTAGAAGGTGACTTTAGGAAGATACTTTAGGAGACTGTAACAGGTAGGGACACAGAGAGACACTCAAGGTAACACCCAAAGACAAACCCAAGGGTCATCCCCAACCCTTAGGGAACCCTCAAGGTCAACCGAAGGTTGACTTTAGGTGATTGGCTGGGCTTTAGGTA‑CCCCTATGGGGGAACTTTAGGTCCGTACAC                              <  1:106622/285‑1`  
                                                                                                                                                                                                                                                                                                    `ggatatgtgtataagaggcaGGTATTTATGTGTAAACTTGAAGATATGCGTTGACTTGATGGGGCTTGAGGGGTAGGCATTAGGTGTTGGCATTAGGATGGACGGTAGGAGGTGACGTTAGGAGGATACTGTATGAGACTGTAACAGATAGGGAAACAGATAGACACTCAAGGTAACAGCCAAAGACAAACCCAAGGGTCATTCTCAACCCTTAGGGAAACCTCAAGGTCAACCGAAGGTTGACTTTAGGTGATTGGCTTGTCTTTAGGTA‑CCCCTATGGGGGAACTTTAGGTCCGTACAC                              <  1:299834/281‑1`  
                                                                                                                                                                                                                                                                                                    `ggagatgtgtataagagacaGGTCTTTATGTTTAAACTTTAAGATAGGCGTTGACTTGATGGGTCTTTAGGTGTAGGCTTTAGGTGTTGTCTTTAGGATGGACGTTAGGAGGTGACTTTAGGAGGATACTTTAGGAGACTGTAACAGATAGGGACACAGAGAGACACTCAAGGTAACACCCAAAGACAAACCCAAGGGTCATCCCCAACCCTTAGGGAACCCTCAAGGTCAACCGAAGGTTGACTTTAGGTGATTGGCTGGGCTTTAGGTA‑CCCCTATGGGGGAACTTTAGGTCCGTACAC                              <  1:453527/281‑1`  
                                                                                                                                                                                                                                                                                                    `gagacaggtTCTTTAGGTCTGGTCTTTATGTTTAAACTTTAAGATAGGCGTTGACTTGATGGGTCTTTAGGTGTAGGCTTTAGGTGTTGGCTTTAGGATGGACGTTAGGAGGTGACTTTAGGAGGATACGTTAGGAGACTGTAACAGATAGGGACACAGAGAGACACTCAAGGTAACACCCAAAGACAAACCCAAGGGTCATCCCCAACCCTTAGGGAACCCTCAAGGTCAACCGAAGGTTGACTTTAGGTGATTGGCTGGGCTTTAGGTA‑CCCCTATGGGGGAACTTTAGGTCCGTACAC                              <  1:76702/292‑1`  
                                                                                                                                                                                                                                                                                                    `agagacagtTCTTTAGGTCTGGTCTTTATGTTTAAACTTTAAGATAGGCGTTGACTTGATGGGTCTTTAGGTGTAGGCTTTAGGTGTTGGCTTTAGGATGGACGTTAGGAGGTGACTTTAGGAGGATACTTTAGGAGACTGTAACAGATAGGGACACAGAGAGACACTCAAGGTAACACCCAAAGACAAACCCAAGGGTCATCCCCAACCCTTAGGGAACCCTCAAGGTCAACCGAAGGTTGACTTTAGGTGATTGGCTGGGCTTTAGGTA‑CCCCTATGGGGGAACTTTAGGTCCGTACAC                              <  1:10353/292‑1`  
                                                                                                                                                                                                                                                                                                    `aaagagacagCTTTAGGTCTGGTCTTTATGTTTAAACGTTAAGATAGGCGTTTACTTGATGGGTCTTTAGGTGTAGGCTTTAGGTGTTGGCTTTAGGATGGACGTTAGGAGGTGACTTTAGGAGGATACTTTAGGAGACTGTAACAGATAGGGACACAGAGAGACACTCAAGGTAGCACCCAAAGACAAACCCAAGGGTCATCCCCAACCCTTAGGGAACCCTCAAGGTCAACCGAAGGTTGACTTTAGGTGATTGGCTGGGCTTTAGGTA‑CCCCTATGGGGGAACTTTAGGTCCATACAC                              <  1:36659/291‑1`  
                                                                                                                                                                                                                                                                                                    `aaagagacaTCTTTAGGTCTGGTCTTTATGTCTAAACTGTAAGATAGGATTTGACTTGATGGGTCTTTAGGTGTATGCTTTAGGTGTTGGCTTTATTATGGACGTTAGGAGGTGACTTTATGAGAATACTTTATGAGACTGTAACAGATAGGGACACAGAGAGACACTCAAGGTAACACCCAAAGACAAACCCAAGGGTCATCCCCAACACTTAGGGAACCCTCAAGGTCAACCGAAGGTTGACTTTAGGTGATTGGCTGGGCTTTAGGTA‑CCCCTATTGGGGAACTTTATGTACGTACAC                              <  1:175236/292‑1`  
                                                                                                                                                                                                                                                                                                            `GTCTTTAGGTCTGGTCTTTATGTTTAAACTTTAAGATAGGCGTTGACTTGATGGGTCTTTAGGTGTAGGCTTTAGGTGTTGGCTTTAGGATGGCCGTTATGAGGTGACTTTAGGAGGATACTTTAGGAGACTGTAACAGATAGGGACACAGAGAGACACTCAAGGTAACACCCAAAGACAAACCCAAGGGTCATCCCCAACCCTTAGGGAACCCTCACGGTCAACCGAAGGTTGACTTTAGGTGATTGGCTGGGCTTTAGGTA‑CCCCTATGGGGGAACTTTAGGTCCGTACACctgtctct                      >  1:323521/1‑293`  
                                                                                                                                                                                                                                                                                                            `GTCTTTAGGTCTGGTCTTTATGTTTAAACTTTAAGATAGGCGTTGACTTGATGGGTCTTTAGGTGTAGGCTTTAGGTGTTGGCTTTAGGATGGACGTTAGGAGGTGACTTTAGGAGGATACTTTAGGAGACTGTAACGGATAGGGACACAGAGAGACACTCAAGGTAACACCCAAAGACAAACCCAAGGGTCATCCCCAACCCTTTGGGAACCCTCAAGGTCACCCGAAGGTTGAATTTAGCATATTGGCTGGGTTTTCGGTA‑CCCCTATGGGGGAACTTTAGGTCCGTACACctgtcttt                      >  1:242159/1‑293`  
                                                                                                                                                                                                                                                                                                            `GTCTTTAGGTCTGGTCTTTATGTTTAAACTTTAAGATAGGCGTTGACTTGATGGGTCTTTAGGTGTAGGCTTTAGGTGTTGGCTTTAGGATGGACGTTAGGAGGTGACTTTAGGAGGATACTTTAGGAGACTGTAACAGATATGGACACAGAGAGACACTCAAGGTAACAACCAAAGACAAACCCAAGGGTCATCCCCAACCCTTATGGAACCCTCAAGGTCAACCGAAGGTTGACTTTAGGTGATTGGCTGGGCTTTAGGTA‑CCTCTATGGGGGAACTTTAGGTCCGTACACctgtctct                      >  1:391633/1‑293`  
                                                                                                                                                                                                                                                                                                            `GTCTTTAGGTCTGGTCTTTATGTTTAAACTTTAAGATAGGCGTTGACTTGATGGGTCTTTAGGTGTAGGCTTTAGGTGTTGGCTTTAGGATGGACGTTAGGAGGTGACTTTAGGAGGATACTTTAGGAGACTGTAACAGATAGGGACACAGAGAGACACTCAAGGTAACACCCAAAGACAAACCCAAGGGTCATCCCCAACCTTTAGGGAACCCTCAAGGTCAACCGAAGGTTGACTTTAGGTGATTGGCTGGGCTTTAGGTA‑CCCCTATGGGGGCACTTTAGGTCCGTACACctgtctct                      >  1:329242/1‑293`  
                                                                                                                                                                                                                                                                                                            `GTCTTTAGGTCTGGTCTTTATGTTTAAACTTTAAGATAGGCGTTGACTTGATGGGGCTTTAGGTGTAGGCTTTAGGTGTTGGCTTTAGGATGGACGTTAGGAGGTGACTTTAGGAGGATCCTTTAGGAGACTGTAACAGATAGGGACACAGAGGGACACTCAAGGTAACACCCAAAGCCAAACCCAAGGGTCATCCCCAACCCTTAGGGAACCCTCCAGGTCAACCGAAGGTTGACTTTAGGTGATTGGCTGGGCTTTAGGTACCCCCTATGGTGGAACTTTGGGTCCGTACACctgtctc                       >  1:189119/1‑294`  
                                                                                                                                                                                                                                                                                                              `CTTTAGGTCTGGTCTTTATGTTTAAACTTTAAGATAGGCGTTGACTTGATGTGTCTTTAGGTGTAGGCTTTAGGTGTTGGCTTTAGGATGGACGTTAGGAGGTGACTTTAGGAGGATACTTTAGGAGACTGTAACAGATAGGGACACAGAGAGACACTCAAGGTAACACCCAAAGACAAACCCAAGGGTCATCCCCAACCCTTAGGGAACCCTCAAGGTCAACCGAAGGTTGACTTTAGGGGATTGGCTGGGCTTTAGGTA‑CCCCTATGTGGGAACTTTAGGTCCGTACACctgtctcttt                    >  1:207086/1‑291`  
                                                                                                                                                                                                                                                                                                              `CTTTAGGTCTGGTCTTTATGTTTAAACTTTAAGATAGGCGTTGACTTGATGGGTCTTTAGGTGTAGGCTTTAGGTGTTGGCTTTAGGATGGACGTTAGGAGGTGACTTTAGGAGGATACTTTAGGAGACTGTAACTGATAGGGACACAGAGAGAAACTCAAGGTAAAACCCAAAGACAAACCCAAGGGTAATCCCCAACCCTTAGGGAACCCTCAAGGTCAACCGAAGGTTGACTTTAGTTGATTGGCTGGGCTTTAGGTA‑CACCTATGGAGGAACGTTCGTTCCGTACACcagtatctta                    >  1:83361/1‑291`  
                                                                                                                                                                                                                                                                                                              `CTTTAGGTCTGGTCTTTATGTTTAAACTTTAAGATAGGCGTTGACTTGATGGGTCTTTAGGTGTAGGCTTTAGGTGTTGGCTTTAGGATGGACGTTAGGAGGTGACTTTAGGAGGATACTTTAGGAGACTGTAACAGATAGGGACACAGAGAGACACTCAAGGTAACACCCAAAGACAAACCCAAGGGTCATCCCCAACCCTTAGGGAACCCTCAAGGTCAACCGAAGGTTGACTTTAGGGGATTGGCTGGGCTTTAGGTACCCCCTATGGGGGAACTTTAGGTCCGTACACctgtccctt                     >  1:274453/1‑292`  
                                                                                                                                                                                                                                                                                                              `CTTTAGGTCTGGTCTTTATGTTTAAACTTTAAGATAGGCGTTGACTTGATGGGTCTTTAGGTGTAGGCTTTAGGTGTTGGCTTTAGGATGGACGTTAGGAGGTGACTTTAGGAGGATACTTTAGGAGACTGTAACAGATAGGGACACAGAGAGACACTCAAGGTAACACCCAAAGACAAACCCAAGGGTCATCCCCAACCCTTAGGGAAACCTCAAGGTCAACCGAAGGTTGACTTTAGGTGATTGGCTGGGCTTTAGGTA‑CCCCTATGGGGGAACTTTAGGTCCGTACctgtctcttata                    >  1:376713/1‑289`  
                                                                                                                                                                                                                                                                                                                  `AGGTCTGGTCTTTATGTTTAAACTTTAAGATAGGCGTTGACTTGATGGGTCTTTAGGTGTAGGCTTTAGGTGTTGGCTTTAGGATGGACGTTAGGAGGTGACTTTAGGAGGATACTTTAGGAGACTGTAACAGATAGGGACACAGAGAGACACTCAAGGTAACACCCAAAGACAAACCCAAGGGTCATCCCCAACCCTTAGGGAACCCTCAAGGTCAACCGAAGGTTGACTTTAGGTGATTGGCTGGGCTTTAGGTA‑CCCCTATGGGGGAACTTTAGGTCCGTACACctgtctcttataca                >  1:406747/1‑287`  
                                                                                                                                                                                                                                                                                                                   `GGTCTGGTCTTTATGTTTAAACTTTAAGATAGGCGTTGACTTGATGGGTCTTTAGGTGTAGGCTTTAGGTGTTGGCTTTAGGATGGACGTTAGGAGGTTACTTTAGGAGGATACTTTAGGAGACTGTAACAGATAGGGACACAGAGAGACACTCAAGGTAACACCCAAAGACAAACCCAAGGGTCATCCCCAACCCTTAGGGAACCCTCAAGGGCAACCGAAGGTTGACTTTAGGTGATTGGCTGGGCTTTAGGTA‑CCCCTATGGGGGAACTTTAGGTCCGTACctgtctcttatacacat               >  1:354698/1‑284`  
                                                                                                                                                                                                                                                                                                                   `GGTCTGGTCTTTATGTTTAAACTTTAAGATAGGCGTTGACTTGATGGGTCTTTAGGTGTAGGCTTTAGGTGTTGGCTTTAGGATGGACGTTAGGAGGTGACTTTAGGAGGATACTTTAGGAGACTGTAACAGATAGGGACACAGAGAGACACTCAAGGTAACACCCACAGACAAACCCAAGGGTCATCCCCAACCCTTAGGGAACCCTCAAGGTCAACCGAAGGTTGACTTTAGGTGATTGGCTGGGCTTTAGGTA‑CCCCTATGGGGGAACTTTAGGTCCtgtctcttatacacatctccg               >  1:119839/1‑280`  
                                                                                                                                                                                                                                                                                                                   `GGTCTGGTCTTTATGTTTAAACTTTAAGATAGGCGTTGACTTGATGGGTCTTTAGGTGTAGGCTTTAGGTGTTGGATTTAGGATGGACGTTAGGAGGTGACTTGAGGAGGATACTTTAGGAGACTGTAACAGATAGGGACACATAGAGACACTCAAGGTAACACCCAAAGACAAACACAAGGGTCATTACCAACCCTTAGGGAACCCTCAAGGTCAACTGAAGGTTGACTTTAGGTGATAGGCTGGGATTTAGGTA‑CCATTTTGGGTGAACTTTCGGTACGTACctgcctcttataaacat               >  1:62120/1‑284`  
                                                                                                                                                                                                                                                                                                                   `GGTCTGGTCTTTATGTTTAAACTTTAAGATAGGCGTTGACTTGATGGGGCTTTAGGTGTAGGCTTTAGGTGTTGGATTTAGGATGGACGTTAGGCGGTGACTTTAGGAGGATACTTTAGGAGACTGTAACAGATAGGGACACAGAGAGACACTCAAGGTAACACCCAAAGACAAACCCAAGGGTCATCCCCAACCCCTGGGGAAACCTCAAGGTCAACCGAAGGTTGACTCAAGGGAATTGGCTGGGCTTTAGGTACCCCCTATGGGGTAACTTTAGGTCCGGACACctgtctctgataca                >  1:287776/1‑287`  
                                                                                                                                                                                                                                                                                                                     `TCTGGTCTTTATGTTTAAACTTTAAGATAGGCGTTGACTTGATGGGTCTTTAGGTGTAGGCTTTAGGTGTTGGCTTTAGGATGGACGTTAGGAGGTGACTTTATGAGGATACCTTAGGAGACTGTAACAGATATGGACACATAGAGACACTCAAGGTAACACCCAAAGACAAACCCAAGGGTCATCCCCAACCCTTAGGGAACCCTCAAGGTCAACCGAAGGTTGACTGTAGGTGATTGGCTGGGCTTTAGGTA‑CCCCTATTGGGGAACTTTAGGTCCGTACAtctcacagtgtacggacc             <  1:381943/301‑19`  
                                                                                                                                                                                                                                                                                                                        `GGTCTTTATGTTTAAACTTTAAGATAGGCGTTGACTTGATGGGTCTTTAGGTGTAGGCTTTAGGTGTTGGCTTTAGGATGGACGTTAGGAGGTGACTTTAGGAGGATACTTTAGGAGACTGTAACAGATAGGGACACAGAGAGACACTCAAGGTAACACCCAAAGACAAACCCAAGGGTAATCCCCAACCCTTAGGGAACCCTCAAGGTCAACCGAAGGTTGACTTTAGGTGATTGGCTGGGCTTTAGGTA‑CCCCTATGGGGGAACTTTAGGTCCtgtctcttatacacatctccgagccc          >  1:153433/1‑275`  
                                                                                                                                                                                                                                                                                                                         `GTCTTTATGTTTAAACTTTAAGATATGCGTTTACTTGATGGGTCTTTAGGTGTAGGCTTTAGGTGTTGGCTTTAGGATGGACGTTAGGAGGTGACTTTAGGAGGATACTTTAGGAGACTGTAACATATAGGGACACAGAGAGACACTCAAGGTAACACACAAAGACAAACCCAAGGGTCGTCCCCAACCCTTAGGGAACCCTCAAGGTCAACCGAAGTTTGACTTTAGGTGATTGGCTGGTCTTTAGGTA‑CCCCTATGGGGGAACTTTAGGTCCGTACctgtctcttttacacatctctga         >  1:171649/1‑278`  
                                                                                                                                                                                                                                                                                                                         `GTCTTTATGTTTAAACTTTAAGATAGGCGTTGACTTGATGGGTCTTTAGGTGTAGGCTTTAGGTGTTGGCTTTAGGATGGACGTTAGGAGGTGACTTTAGGAGGATACTTTAGGAGACTGTAACAGATAGGGACACAGAGAGACACTCAAGGTAACACCCAAAGACAAACCCATGGGTCATCCCCAACCCTTAGGGAACCCTCAAGGTCAACCGAAGGTTGACTTTAGGTGATTGGCTGGGCTTTAGGTA‑CCCCTATGGGGGAACTTTAGGTCCGTACACctgtctcttatacacatctcc         >  1:6963/1‑280`  
                                                                                                                                                                                                                                                                                                                               `ATGTTTAAACTTTAAGATAGGCGTTGACTTGATGGGTCTTTAGGTGTAGGCTTTAGGTGTTGGCTTTAGGATGGACGTTAGGAGGTGACTTTAGGAGGATACTTTAGGAGACTGTAACATATAGGGACACAGAGAGACACTCAAGGTAACACCCAAAGACAAACCCAAGGGTCATCCCCAACCCTTAGGGAACCATCAAGGTCAACCGAAGGTTGACTTTAGGTGATTGGCTGGGCTTTAGGTA‑CCCCTATGGGGGAACTTTAGGTCCATAC‑CTGTGtcttatacacatctccgagcccac  >  1:435905/1‑277`  

                                                                                                                                                                                                                                                                                                                                                                                                                                                                                                                                                                                                                                                
`TACTGCATGTTACTTCATGTTGTCAATACCTGTTTTTCGTGCGACTTATCAGGCTGTCTACTTATCCGGTTGACCCCGGTGTCGATTGGGATGGCTATTCGCCGTGTCCCTCTCGATGGCTGTAAGTATCCTATAGGTTAGACTTTAAGTCAATACTCTTTTTGATAAATTTTAAATTAATCTTTTAAGTCTCTTTAAGTTGTCTCTCCTTATAGTGAGTTGTATTAATAAAGACAATGATTAAAGGTTATCACTTTATGGCTAACAACAAGGCGTCTTTAGGTCTGGTCTTTATGTAGTGTCTTTAGGTCTGGTCTTTA‑‑‑‑‑‑‑‑‑‑‑‑‑‑‑‑‑‑‑‑‑‑‑‑‑‑‑‑‑‑‑‑‑‑‑‑‑‑‑‑‑‑‑‑‑‑‑‑‑‑‑‑‑‑‑‑‑‑‑‑‑‑‑‑‑‑‑‑‑‑‑‑‑‑‑‑‑‑‑‑‑‑‑‑‑‑‑‑‑‑‑‑‑‑‑‑‑‑‑‑‑‑‑‑‑‑‑‑‑‑‑‑‑‑‑‑‑‑‑‑‑‑‑‑‑‑‑‑‑‑‑‑‑‑‑‑‑‑‑‑‑‑‑‑‑‑‑‑‑‑‑‑‑‑‑‑‑‑‑‑‑‑‑‑‑‑‑‑‑‑‑‑‑‑‑‑‑‑‑‑‑‑‑‑‑‑‑‑‑‑‑‑‑‑‑‑‑‑‑‑‑‑‑‑‑‑‑‑‑‑‑‑‑‑‑‑‑‑‑‑‑‑‑‑‑‑‑‑‑‑‑‑‑‑‑‑‑‑‑‑‑‑‑‑‑‑‑‑‑‑‑‑‑‑‑‑‑‑‑‑‑‑‑‑‑‑‑‑‑‑‑‑‑‑‑‑‑‑‑‑‑‑‑‑‑‑‑‑‑‑‑‑‑‑‑‑‑‑‑‑‑‑  <  NC_001604.1/614‑295`  
`‑‑‑‑‑‑‑‑‑‑‑‑‑‑‑‑‑‑‑‑‑‑‑‑‑‑‑‑‑‑‑‑‑‑‑‑‑‑‑‑‑‑‑‑‑‑‑‑‑‑‑‑‑‑‑‑‑‑‑‑‑‑‑‑‑‑‑‑‑‑‑‑‑‑‑‑‑‑‑‑‑‑‑‑‑‑‑‑‑‑‑‑‑‑‑‑‑‑‑‑‑‑‑‑‑‑‑‑‑‑‑‑‑‑‑‑‑‑‑‑‑‑‑‑‑‑‑‑‑‑‑‑‑‑‑‑‑‑‑‑‑‑‑‑‑‑‑‑‑‑‑‑‑‑‑‑‑‑‑‑‑‑‑‑‑‑‑‑‑‑‑‑‑‑‑‑‑‑‑‑‑‑‑‑‑‑‑‑‑‑‑‑‑‑‑‑‑‑‑‑‑‑‑‑‑‑‑‑‑‑‑‑‑‑‑‑‑‑‑‑‑‑‑‑‑‑‑‑‑‑‑‑‑‑‑‑‑‑‑‑‑‑‑‑‑‑‑‑‑‑‑‑‑‑‑‑‑‑‑‑‑‑‑‑‑‑‑‑‑‑‑‑‑‑‑‑‑‑‑‑‑‑‑‑‑‑‑‑‑‑‑‑‑‑‑‑‑‑‑‑gtctttaggtctggtctttaTGTTTAAACTTTAAGATAGGCGTTGACTTGATGGGTCTTTAGGTGTAGGCTTTAGGTGTTGGCTTTAGGATGGACGTTAGGAGGTGACTTTAGGAGGATACTTTAGGAGACTGTAACAGATAGGGACACAGAGAGACACTCAAGGTAACACCCAAAGACAAACCCAAGGGTCATCCCCAACCCTTAGGGAACCCTCAAGGTCAACCGAAGGTTGACTTTAGGTGATTGGCTGGGCTTTAGGTACCCCCTATGGGGGAACTTTAGGTCCGTACACTGTG‑‑‑‑‑‑‑‑‑‑‑‑‑‑‑‑‑‑‑‑‑AGA  <  NC_001604.1/281‑1`

  

Alignment Legend

Aligned base mismatch/match (shaded by quality score): `ATCG/ATCG` `< 3 ≤` `ATCG/ATCG` `< 10 ≤` `ATCG/ATCG` `< 18 ≤` `ATCG/ATCG` `< 33 ≤` `ATCG/ATCG` `< 38 ≤` `ATCG/ATCG`

Unaligned base: `atcg`    Masked matching base: `atcg`    Alignment gap: `‑`    Deleted base: `‑`

Reads not counted as support for junction

read\_name Not counted due to insufficient overlap past the breakpoint.

read\_name Not counted due to not crossing MOB target site duplication.
